# Migration-associated malaria from Africa in southern Spain

**DOI:** 10.1186/s13071-021-04727-0

**Published:** 2021-05-07

**Authors:** Joaquín Pousibet-Puerto, Ana Belén Lozano-Serrano, Manuel Jesús Soriano-Pérez, José Vázquez-Villegas, María José Giménez-López, María Isabel Cabeza-Barrera, José Ángel Cuenca-Gómez, Matilde Palanca-Giménez, María Pilar Luzón-García, Nerea Castillo-Fernández, María Teresa Cabezas-Fernández, Joaquín Salas-Coronas

**Affiliations:** 1 Tropical Medicine Unit. Hospital de Poniente, El Ejido, Almería Spain; 2 Tropical Medicine Unit. Distrito Poniente, Almería, Spain; 3 Hematology Unit. Hospital de Poniente, El Ejido, Almería Spain; 4 CEMyRI (Center for the Study of Migration and Intercultural Relations) of the University of Almeria, Almería, Spain

**Keywords:** Malaria, Immigrants, Sub-Saharan Africa, VFR migrants, *P. falciparum*, Submicroscopic malaria, Semi-immunity, Coinfections

## Abstract

**Background:**

The western area of the province of Almeria, sited in southern Spain, has one of the highest immigrant population rates in Spain, mainly dedicated to agricultural work. In recent years, there has been a significant increase in the number of cases of imported malaria associated with migrants from countries belonging to sub-Saharan Africa. The objective of our study is to describe the epidemiological, clinical and analytical characteristics of malaria patients treated in a specialized tropical unit, paying special attention to the differences between VFR and non-VFR migrants and also to the peculiarities of microscopic malaria cases compared to submicroscopic ones.

**Methods:**

Retrospective observational study of migrants over 14 years of age with imported malaria treated from October 2004 to May 2019. Characteristics of VFR and non-VFR migrants were compared. Malaria cases were divided into microscopic malaria (MM) and submicroscopic malaria (SMM). SMM was defined as the presence of a positive malaria PCR test together with a negative direct microscopic examination and a negative rapid diagnostic test (RDT). Microscopic malaria was defined as the presence of a positive RDT and/or a positive smear examination.

**Results:**

Three hundred thirty-six cases of malaria were diagnosed, 329 in sub-Saharan immigrants. Of these, 78.1% were VFR migrants, in whom MM predominated (85.2% of cases). In non-VFR migrants, SMM represented 72.2% of the cases. Overall, 239 (72.6%) patients presented MM and 90 (27.4%) SMM. Fever was the most frequent clinical manifestation (64.4%), mainly in the MM group (MM: 81.1% *vs* SMM: 20.0%; *p* < 0.01). The most frequent species was *P. falciparum*. Patients with SMM presented fewer cytopenias and a greater number of coinfections due to soil-transmitted helminths, filarial and intestinal protozoa compared to patients with MM.

**Conclusions:**

Imported malaria in our area is closely related to sub-Saharan migration. VFR migrants are the main risk group, highlighting the need for actions aimed at improving disease prevention measures. On the other hand, almost a third of the cases are due to SMM. This fact could justify its systematic screening, at least for those travelers at greater risk.

**Graphic Abstract:**

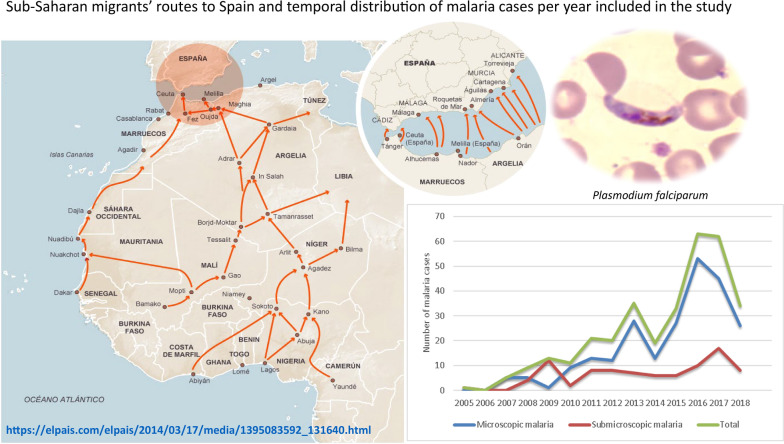

## Background

Malaria is still the parasitic disease with the highest morbimortality worldwide, although its incidence has decreased during the last decade. In 2018, the World Health Organization (WHO) declared a total of 228 million of malaria cases globally with 93% of them occurring in sub-Saharan Africa [1]. In Europe, around 8000 cases of imported malaria are reported every year, the majority due to *P. falciparum* [2]. Around 90% of them come from sub-Saharan Africa and are mainly diagnosed in newly arrived migrants or in resident migrants traveling back to their home-countries to visit friends and relatives (VFR, visiting friends and relatives). In this last group of travelers, risk perception for malaria is low and preventive measures are usually not followed [3–6].

Although most of the cases reported in Europe are patients with microscopic malaria (MM), submicroscopic malaria (SMM) [7–9] is also present. SMM may reach a prevalence of near 9% in asymptomatic patients from endemic areas [7] even though figures are probably underestimated because of the absence of symptoms and the fact that molecular diagnosis is not routinely employed.

In Spain, a mean 700–800 cases of imported malaria are declared every year [10]. The Poniente area (province of Almeria, Spain) is an administrative health area sited in southern Spain. Its population is close to 300,000 inhabitants, with an immigrant share of 21%, many of them coming from sub-Saharan countries to work in horticultural greenhouses [11]. Malaria is one of the most prevalent imported diseases in our area [7, 12], and the numbers have increased in recent years parallel to the growing migratory phenomenon.

Spain was granted the official WHO certification of malaria elimination in 1964 after the last case of autochthonous malaria was reported in 1961 [13]. Since then, malaria has been considered an imported disease, although a situation of anophelism without malaria persists. Three species of *Plasmodium* were endemic in Spain, *P. falciparum, P. malariae* and *P. vivax*, while there were no reports of the presence of *P. ovale*. The vectors of the disease belonged to the *Anopheles maculipennis* complex, the main ones being *An. atroparvus* and *An. labranchiae*, the latter with marked antropophilia. Although *An. atroparvus* is widely distributed in Spain, the presence of *An. labranchiae* in our country is not as evident nowadays. Nevertheless, it continues to be abundantly present in northern Morocco [14]; thus, its reintroduction, either naturally transported by the wind [15] or inadvertently transported inside vehicles, is possible and a definite risk to be considered. Two cases of autochthonous malaria due to vector transmission were reported in 2010 and 2014 [16, 17]. In both cases, *P. vivax* was the species involved.

The objective of our study is to describe the cases of imported malaria diagnosed in our area during the last 15 years. Demographical, clinical and analytical characteristics are pinpointed paying special attention to the differences between VFR and non-VFR migrants.

## Methods

A retrospective observational study was conducted using the data obtained from the registry of migrant patients assisted at the Tropical Medicine Unit (TMU) of the Poniente Hospital (El Ejido, Almeria, Spain) from October 2004 to May 2019. All migrant patients > 14 years of age diagnosed with malaria during that period were included in the study.

Migrants were classified as VFR or non-VFR migrants. Spain-based migrants traveling to their homeland to visit friends and relatives were considered VFR, whereas those migrants traveling for the first time to Europe from malaria-endemic areas were considered non-VFR migrants.

Malaria diagnosis was made by means of rapid direct tests, direct microscopic tests and/or molecular tests. In all cases, the techniques were performed using peripheral venous blood samples extracted in 5-ml EDTA tubes.

A screening test for SMM malaria was offered to all travelers coming back from an endemic malaria area if they consulted within the first 12 months after the travel, no matter the reason they consulted for. A conventional Nested Multiplex Malaria PCR (NM-PCR) capable of identifying four human malaria species (*P. vivax, P. falciparum, P. ovale* and *P. malariae*) was used [18]. If this PCR test yielded a positive result, MM was then ruled out following the same scheme described for patent malaria suspicious cases (see below).

However, all those patients presenting with symptoms suggesting patent malaria, irrespective of the time elapsed after leaving a malaria-endemic area, were tested by immunochromatographic rapid diagnostic test (RDT, SD BiolineR malaria Ag Pf/Pan, Korea) and direct microscopic examination of thin smear. In these symptomatic cases, NM-PCR was only used when a mixed infection was suspected or when direct microscopy and RDT were negative but a high clinical suspicion persisted.

Therefore, the malaria cases included in our study comprised submicroscopic malaria (SMM) and of microscopic malaria (MM). SMM was defined as the presence of a positive malaria PCR test together with a negative direct microscopic examination and a negative RDT. Microscopic malaria was defined as the presence of a positive RDT and/or a positive smear examination. For cases of MM, the parasitemia level was classified into four categories: ≤ 1, > 1–2.5, > 2.5–4% and > 4%.

In addition, and following our healthcare protocol for migrants, all sub-Saharan patients attending our TMU are offered a comprehensive screening battery for imported diseases including chest and abdominal x-rays, blood count, liver and renal function tests, iron metabolism, serological tests for syphilis, HIV, HBV, HCV, *Strongyloides* and *Schistosoma*, tuberculin test, and search for stool parasites, urine parasites, and blood microfilariae. Hemoglobinopathies are also ruled out by HPLC (high-performance liquid chromatography).

Statistical analyses were performed using the Statistical Package for the Social Sciences (SSPS) version 17. A descriptive analysis of all patients included in the study was made. Quantitative variables were expressed as median and interquartile range. For qualitative variables, absolute and relative frequencies were calculated. Comparative analysis between VFR and non-VFR migrants and between MM and SMM cases were also made. Differences in continuous variables between groups were analyzed using Students’s t-test or the Mann-Whitney U test, as appropriate. To compare categorical variables, we used the Fisher test or the chi-square test. A *p*-value < 0.05 was considered statistically significant for all of the tests.

### Ethics approval

Anonymized data were collected retrospectively. This study was approved by the local Ethics Committee of the Coordinating Site (Almería, Spain) with the code MT-02-2020.

## Results

During the study period, a total of 336 cases of imported malaria were found. Seven of them occurred in Spanish native patients and were excluded from the study. The rest, 329 cases, occurred in sub-Saharan migrants, the majority of them being VFR (78.1%) and male (90.9%). *P. falciparum* was the most frequently found plasmodium species (92.1%).

**Fig. 1:**
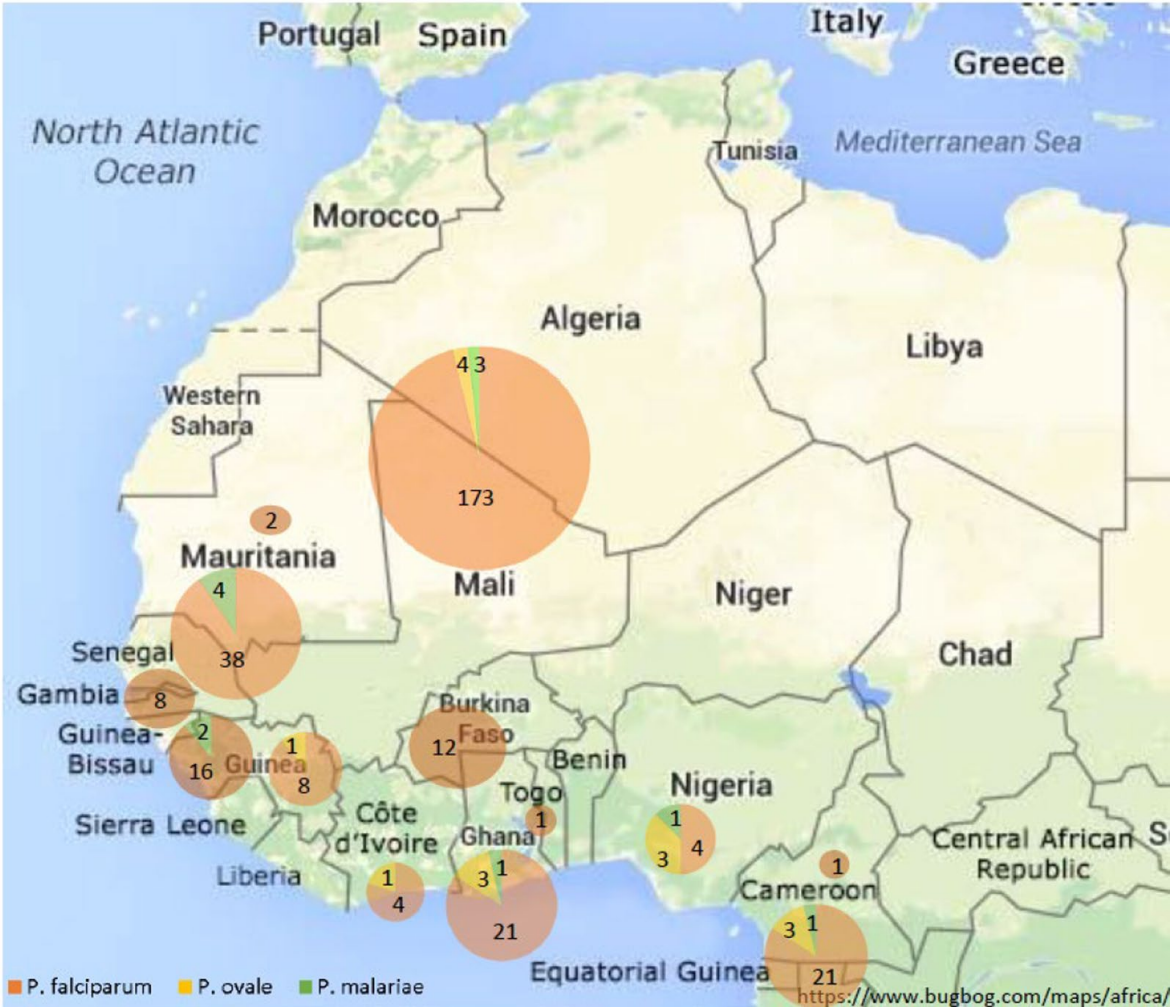
Distribution of imported malaria cases including mixed infections

Demographics and general characteristics of the patients, as a whole and divided into VFR and non-VFR groups, are shown in Table 1. The main countries of origin were Mali (53.8%), Senegal (12.8%) and Equatorial Guinea (7.6%) (Fig. 1). Male gender predominated in both groups. The median time of residence in Spain for VFR migrants was 118 months.

**Table 1:** Epidemiological, clinical and analytical characteristics of patients included in the study, grouped by type of traveler

	All N = 329	VFR 257 (78.1%)	Non VFR 72 (21.9%)	*p* value
Age (years)	33 (11)	35.17 (7.83)	29.96 (12.06)	< 0.001
Gender (%)				
Male	299 (90.9%)	240 (93.4%)	59 (81.9%)	0.003
Female	30 (9.1%)	17 (6.6%)	13 (18.1%)	
Time after leaving malaria-endemic areas (%)				
≤ 12 months	316 (96.0%)	253 (98.4%)	63 (87.5%)	0.011
> 12 months	13 (4.0%)	4 (1.6%)	9 (12.5%)	
Country of origin (%)				
Mali	177 (53.8%)	157 (61.1%)	20 (27.8%)	
Senegal	42 (12.8%)	31 (12.1%)	11 (15.3%)	
Equatorial Guinea	25 (7.6%)	11 (4.3%)	14 (19.4%)	
Ghana	24 (7.3%)	19 (7.4%)	5 (6.9%)	
Guinea Bissau	18 (5.5%)	12 (4.7%)	6 (8.3%)	
Burkina-Faso	12 (3.6%)	5 (1.9%)	7 (9.7%)	
Gambia	8 (2.4%)	6 (2.3%)	2 (2.8%)	
Guinea-Conakry	8 (2.4%)	6 (2.3%)	2 (2.8%)	
Nigeria	7 (2.1%)	5 (1.9%)	2 (2.8%)	
Ivory Coast	4 (1.2%)	2 (0.8%)	2 (0.8%)	
Mauritania	2 (0.6%)	2 (0.8%)	0 (0.0%)	
Cameroon	1 (0.3%)	1 (0.4%)	0 (0.0%)	
Togo	1 (0.0%)	0 (0.0%)	1 (1.4%)	
Symptoms (%)				
Fever	212 (64.4%)	202 (78.6%)	10 (13.9%)	< 0.001
Diarrhea	9 (2.7%)	8 (3.1%)	1 (1.4%)	0.428
Abdominal pain	49 (14.9%)	33 (12.8%)	16 (22.2%)	0.048
Others/asymptomatic	34 (10.3%)	13 (5.1%)	21 (29.2%)	< 0.001
MM (%)	239 (72.6%)	219 (85.2%)	20 (27.8%)	< 0.001
SMM (%)	90 (27.4%)	38 (14.8%)	52 (72.2%)	
*Plasmodium* species (%)				
* P. falciparum*	303 (92.1%)	245 (95.3%)	58 (80.6%)	< 0.001
* P. ovale*	10 (3.0%)	3 (1.2%)	7 (9.7%)	
* P. malariae*	9 (2.7%)	4 (1.6%)	5 (6.9%)	
Mixed infections:	7 (2.1%)	5 (1.9%)	2 (2.8%)	
* P. falciparum* + *P. malariae*	3	2	1	
* P. falciparum* + *P. ovale*	3	2	1	
*P. malariae* + *P. ovale*	1	1		
Parasitemia (%)				
< 1	106 (44.3%)	92 (43%)	14 (87.5%)	< 0.001
1–2.5	65 (27.2%)	64 (29.9%)	1 (6.3%)	
2.5–4	20 (8.4%)	20 (9.3%)	0 (0%)	
> 4	39 (16.3%)	38 (17.8%)	1 (6.3%)	
Not available	9 (3.8%)			

*MM* microscopic malaria. *SMM* submicroscopic malaria. *VFR* Visiting friends and relatives. Quantitative variables: values are median (interquartile range, IQR). Qualitative variables: values are number (percentage), *N* (%)

Microscopic malaria was the most frequent form of malaria for VFR migrants (85.2%). Conversely, it represented only 27.8% of the malaria cases for non-VFR migrants, for whom submicroscopic malaria was predominant. When presenting as MM, most non-VFR migrants had low levels of parasitemia compared to less than half of the VFR migrants (87.5 *vs* 43% *p* ≤ 0.01). Moreover, of the 39 patients with parasitemia > 4%, all but one belonged to the VFR group.

The characteristics of imported malaria comparing MM against SMM are shown in Table 2. Of the 329 cases of malaria, 239 (72.6%) were MM and 90 (27.4%) SMM. Most of the MM cases, 91.6%, occurred in VFR migrants while SMM cases were divided more evenly between VFR and non-VFR. Only 8.9% (*n* = 23) of VFR migrants reported taking malaria prophylaxis properly.

**Table 2:** Epidemiological, clinical and analytical characteristics of patients included in the study, grouped by type of malaria

	MM	SMM	*p* value
	239 (72.6%)	90 (27.4%)	
Age (years)	34 (10)	31 (14)	≤ 0.01
Time after leaving malaria-endemic areas (%)			
≤ 12 months	234 (97.9%)	82 (91.1%)	≤ 0.01
> 12 months	5 (2.1%)	8 (8.9%)	
Gender (%)			
Male	222 (92.9%)	77 (85.6%)	0.04
Female	17 (7.1%)	13 (14.4%)	
Type of traveler			
VFR	219 (91.6%)	38 (42.2%)	≤ 0.01
Non-VFR	20 (8.4%)	52 (57.8%)	≤ 0.01
Symptoms (%)			
Fever	194 (81.1%)	18 (20.0%)	≤ 0.01
Diarrhea	9 (3.8%)	0 (0.0%)	0.12
Abdominal pain	31 (13.0%)	18 (20.0%)	0.11
Others/asymptomatic	5 (2.1%)	29 (32.2%)	≤ 0.01
*Plasmodium* species (%)			
* P. falciparum*	229 (95.8%)	74 (82.2%)	≤ 0.01
* P. ovale*	4 (1.7%)	6 (6.7%)	
* P. malariae*	1 (0.4%)	8 (8.9%)	
Mixed infections	5 (2.1%)	2 (2.2%)	
* P. falciparum* + *P. malariae*	1	2	
* P. falciparum* + *P. ovale*	3		
* P. malariae* + *P. ovale*	1		
Malaria chemoprophylaxis in VFR travelers (%)			
No	135 (61.6%)	21 (55.3%)	0.67
Yes	15 (6.8%)	8 (21.0%)	
Inadequate or suboptimal	22 (10.0%)	2 (5.3%)	
Unknown	47(21.5%)	7 (18.4%)	

Quantitative variables: values are median (interquartile range, IQR). Qualitative variables: values are number (percentage), *N* (%)

*MM* microscopic malaria, *SMM* submicroscopic malaria, *VFR* visiting friends and relatives

Globally, fever was the most frequent clinical sign, present in 81.1% of all MM cases but in only 20.0% of the SMM cases (*p* ≤ 0.01). In this former group, abdominal pain was as frequent as fever. *Plasmodium falciparum* was the predominant species (*n* = 303; 92.1%), followed by *P. ovale* (*n* = 10, 3%) and *P. malariae* (*n* = 9, 2.7%). In seven cases, a mixed parasitization was found. Around one third of MM cases (32.2%) had a parasitemia lower than 1%.

Most of the cases of malaria included in our study were diagnosed within the first weeks or months after arriving from endemic areas. Only 13 patients were diagnosed after > 12 months after the trip, 9 of them with *P. falciparum*, 3 with *P. malariae* and 1 with *P. ovale*. Five of them presented with MM and eight with SMM. All five patients with MM reported symptoms (mainly fever) or showed laboratory abnormalities suggesting malaria. Among the eight patients presenting with SMM, five also had some findings compatible with malaria, either splenomegaly or cytopenias. The other three patients who were offered a PCR test for malaria beyond the 12 months limit were asymptomatic for malaria. In these three cases, malaria testing was ordered by the clinician in charge of the patients without following the usual protocol. As a matter of fact, most patients with a positive PCR screening in our study had arrived to Spain in the last 6 months or less (72.2% in the last month, 85.6% in the last 6 months).

Laboratory tests results are displayed in Table 3. Hemoglobin concentration, platelet and eosinophil counts were lower in patients with MM compared to patients with SMM, whereas bilirubin levels were higher. On the contrary, the distribution of hemoglobinopathies was similar between the groups.

Associated co-infections are described in Table 4. Briefly, the presence of geohelminths, filariae and intestinal protozoa was higher in the SMM group, probably related to the greater eosinophil counts found in this group. All patients were treated with quinine sulfate plus doxycycline, cloroquine plus primaquine, atovaquone-proguanil or dihydroartemisin-piperaquine according to the WHO guidelines in force at the time. Clinical evolution was favorable in every case, and there were no deaths in the series.

**Table 3:** Laboratory tests results for all patients included in the study, grouped by type of malaria

	ALL	MM	SMM	*p* value
	*N* = 329	239 (72.6%)	90 (27.4%)	
Hb (g/dl)	13.6 (2.2)	13.3 (2.0)	14.3 (2.8)	≤ 0.01
Platelets × 10^3^/µl	114 (130)	90 (86)	216 (95)	≤ 0.01
Total bilirubin (mg/dl)	1.1(1.1)	1.3 (1.4)	0.7 (0.6)	≤ 0.01
Structural hemoglobinopathies				
No	237 (72.0%)	175 (76.7%)	62 (70.4%)	0.25
Yes	79 (24.0%)	53 (22.2%)	26 (29.5%)	
Not available	13 (3.9%)			

Quantitative variables: Median (interquartile range, IQR); quantitative variables: values are number (percentage), *N* (%)

*MM* microscopic malaria, *SMM* submicroscopic malaria, *HB* Hemoglobin

**Table 4:** Co-infections found in the patients included in the study, grouped by type of malaria

Co-infection *N* (%)	ALL	MM	SMM	*p* value
	N = 329	239 (72.6%)	90 (27.4%)	
*Blastocystis hominis*	40 (12.1%)	20 (8.4%)	20 (22.2%)	≤ 0.01
*Entamoeba hystolitica/dispar*	15 (4.5%)	6 (2.5%)	9 (10.0%)	≤ 0.05
*Giardia lamblia*	7 (2.1%)	5 (2.1%)	2 (2.2%)	1
*Strongyloides stercoralis*	31 (9.4%)	16 (6.7%)	15 (16.7%)	≤ 0.05
Hookworms	10 (3.0%)	3 (1.3%)	7 (7.8%)	≤ 0.01
*Trichuris trichiura*	2 (0.6%)	1 (0.4%)	1 (1.1%)	0.54
*Ascaris lumbricoides*	3 (0.9%)	1 (0.4%)	2 (2.2%)	0.25
*Hymenolepsis nana*	3 (0.9%)	0 (0.0%)	3 (3.3%)	≤ 0.05
Schistosomiasis*	22 (6.7%)	14 (5.9%)	8 (8.9%)	0.629
*S. haematobium*	6 (1.8%)	3 (1.3%)	3 (3.3%)	0.37
*S. mansoni*	6 (1.8%)	3 (1.3%)	3 (3.3%)	0.39
*S. intercalatum*	1 (0.3%)	0 (0.0%)	1 (1.1%)	0.32
*Schistosoma* spp.	5 (1.5%)	4 (1.7%)	1 (1.1%)	0.3
Filariae	14 (4.2%)	4 (1.7%)	10 (11.1%)	≤ 0.01
*Mansonella perstans*	13 (3.9%)	2 (0.8%)	9 (10.0%)	≤ 0.01
*Loa loa*	3 (0.9%)	0 (0.0%)	3 (3.3%)	≤ 0.05
Syphilis	23 (7.0%)	11 (4.6%)	12 (13.3%)	≤ 0.01
HBV	49 (14.9%)	24 (10.0%)	25 (27.8%)	≤ 0.01
HCV	2 (0.6%)	1 (0.4%)	1 (1.1%)	0.39
HIV	10 (3.0%)	9 (3.8%)	1 (1.1%)	0.29

Quatitative variables: values are number (percentage), *N* (%).* Probable schistosomiasis was diagnosed in four patients based on clinical, analytical and imaging tests although no schistosome was found

*MM* microscopic malaria, *SMM* submicroscopic malaria, *HBV* hepatitis B virus, *HCV* hepatitis C virus, *HIV* human immunodeficiency virus

**Fig. 2:**
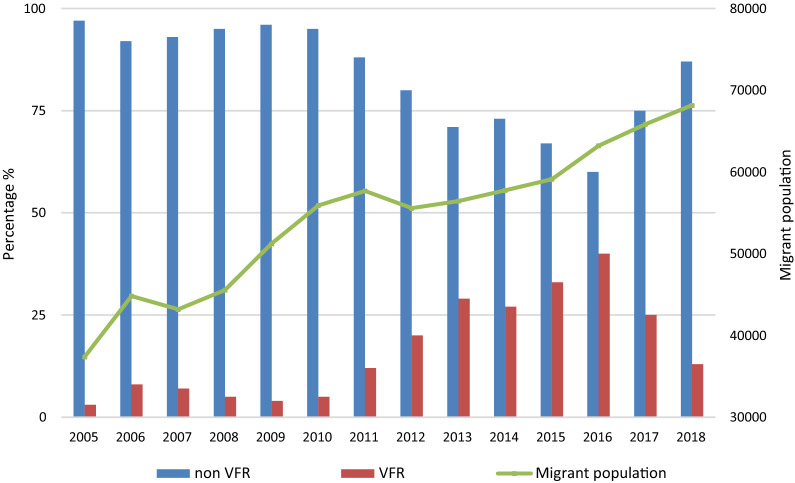
Temporal evolution of migration in our area during the study period. VFR: visiting friends and relatives

**Fig. 3:**
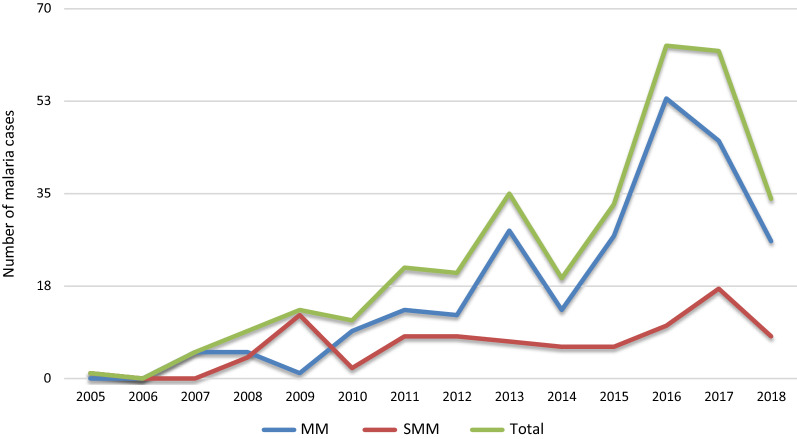
Temporal distribution of malaria cases per year. MM: microscopic malaria. SMM: submicroscopic malaria

**Fig. 4:**
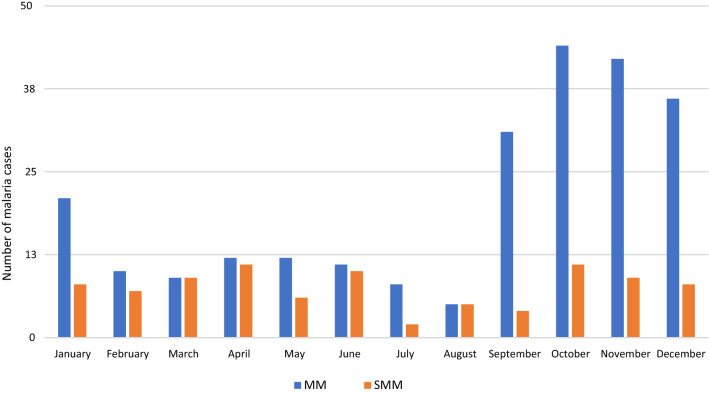
Monthly distribution of the malaria cases included in the study. MM: microscopic malaria. SMM: submicroscopic malaria

Figure 2 shows the temporal evolution of the African migrant population in the province of Almeria, according to data from the Statistics National Institute, during the study period. The progressive increase of this population has been followed by an increasing proportion of VFR migrants attending our unit as given in Fig. 3, which shows a continuous increment in the number of malaria cases per year. The majority of cases were concentrated in the last months of the year (Fig. 4), the time when VFR migrants traveled back to Spain after spending the holidays in their home countries.

## Discussion

Our study explores the imported malaria cases occurring during the last 15 years in our area of influence, a setting with high migration rates from sub-Saharan Africa. The results show a progressive increment of malaria cases parallel to the growing migration phenomenon. Imported malaria cases are mostly due to sub-Saharan migrants coming to our area looking for a jobs in the agricultural industry, which flourishes under the greenhouses. Most of the cases are related to VFR migrants rather than to newly arrived migrants (non-VFR).

VFR migrants represent up to 50% of international travelers coming from developed countries [19]. They are a population with a special risk of acquiring infectious diseases during the trip. Compared to other travelers, such as tourists, they have a higher risk of contracting malaria, hepatitis A and B, sexually transmitted infections and intestinal parasites [6]. GeoSentinel places sub-Saharan Africa as the main area for acquiring malaria (83% of cases) and the subgroup of VFR migrants as the group of travelers with the highest risk (53% of total cases) [6]. In Spain, data from + REDIVI, a cooperative network for the study of diseases imported by immigrants and travelers, show that VFR accounts for 58% of malaria cases and that > 95% are acquired in sub-Saharan Africa [20]. In our study, the percentage of VFR is 78% considering only the migrant population, and, including all kind of travelers (expatriates, tourists, etc.), it represents 76% of total imported malaria cases. Accordingly, most of the cases are diagnosed after the summer holidays, when migrants return from visiting their home countries to get back to work.

Other aspects observed in our study in relation to VFR migrants is that MM was the predominating form of malaria and that *P. falciparum* was the species most frequently found. These data correspond with those previously reported in Europe [4–8, 10, 12, 19–21]. In the + REDIVI study, *P. falciparum* was also the most frequent species (81.5% of all cases; 86% in the VFR subgroup) [20].

Although > 15% of the cases in the VFR group had parasitemia > 4%, a well-known severity criterion, no deaths ocurred. This is probably due to several reasons: (i) VFR migrants retain a certain degree of semi-immunity despite having left malaria-endemic regions long ago; (ii) health staff working at the Emergency Room or at Primary Care in our area are well trained in recognizing malaria symptoms so diagnosis is not delayed or missed; (iii) the availability of the entire therapeutic arsenal needed to quickly care for malaria patients in our center.

The profile of malaria cases in non-VFR migrants is different, with SMM being the predominant form of malaria. Diagnosis of SMM is largely due to the systematic screening applied in our unit to those patients that have visited or left from a malaria-endemic area in the last 12 months [7]. Limitation of SMM screening to such time interval is due to the poorer performance of the screening strategy beyond that limit. In our experience (unpublished data), out of 213 asymptomatic migrants screened for malaria using a PCR test after having left malaria-endemic areas > 12 months ago, only 3 (1.4%) yielded a positive result. In our study, 13 patients were diagnosed 12 months or more after leaving malaria-endemic areas, the majority of them being due to *P. falciparum* and presenting as SMM. The exact mechanism allowing the persistence of the parasite for months, or even years, without causing clinically overt disease is not entirely well known, though several strategies aimed to evade the immune response have been described [22].

As we have observed in this study, SMM tends to be asymptomatic and presents fewer cytopenias than MM [8, 23, 24], although symptomatic reactivations may be possible several years after leaving malaria-endemic areas [22].

In endemic areas where the infection rate is low or unstable, SMM plays an important role in the transmission of malaria and poses a problem for its eradication [25]. In non-endemic areas, these SMM asymptomatic infections can act as a source for the reintroduction of malaria. Their exact prevalence is unknown, but they may account for one third of imported malaria cases in Europe [7, 8]. In Spain, *Anopheles atroparvus* mosquitoes can transmit *P. vivax* strains [26], and, indeed, two cases of locally acquired *P. vivax* malaria have been reported in recent years, as previously mentioned [16, 17]. Moreover, due to the proximity to Morocco, where *An. labranchiae* is present [14], the risk of *P. falciparum* malaria is also a threat.

SMM usually affects individuals considered semi-immune who have lived for a long time in highly endemic areas. This partial immunity is gradually acquired by prolonged exposure to *Plasmodium* spp. as individuals grow older. Once out of endemic regions, the exact duration of this protection against malaria is unknown, but semi-immunity decreases as time living in the host country increases [9, 19, 21, 27]. Studies in non-endemic countries comparing travelers with different degrees of previous exposure to the malaria parasite are scarce, but a greater protection against severe forms has been observed in VFR migrants compared to European travelers [8, 28, 29]. For these reasons, and although we can consider that all the patients included in our study have some degree of semi-immunity, this protection may be weaker in the case of VFR migrants. This fact could explain the higher proportion of MM diagnosed in this group as well as the higher parasite loads compared to non-VFR migrants, who have been living out of malaria-endemic areas for < 1 year in many cases.

Given that in our present study > 25% of the patients presented SMM, and although cost-effectiveness studies are lacking, universal malaria screening strategies by means of molecular tests could be considered for asymptomatic migrants coming from endemic regions [7, 8, 30, 31]. This screening might be especially recommended for vulnerable travelers such as pregnant women and immunosuppressed individuals, who are more prone to suffer serious consequences from an undiagnosed SMM.

In the analysis of co-infections, the higher proportion of soil-transmitted helminths and filariae in the group of travelers with SMM is worth mentioning. This fact could partly explain the higher levels of eosinophils in the former compared to the MM group. In tropical and subtropical regions, especially in sub-Saharan Africa, polyparasitism is highly prevalent. Nematodes are able to attenuate the response of inflammatory molecules such as cytokines or interleukins of their hosts to survive for long periods. However, this immunomodulatory effect can also modify the response to concomitant intracellular infections such as tuberculosis or malaria [32–35]. In particular, filarial coinfection appears to exert some protection against severe malaria by modulating certain interleukins and IFN-γ [36, 37]. This protective effect can lead to lower parasite loads as in SMM, fewer cytopenias and fewer complications associated with malaria [37, 38]. In non-endemic areas, a higher proportion of filarial co-infections has also been observed in patients with SMM compared to MM patients [7, 8]. Prospective studies in endemic and non-endemic areas are needed to verify this hypothesis. In the meantime, ruling out SMM could be recommended for migrants presenting with filarial infections, although these patients are probably less likely to develop serious symptomatic malaria.

The limitations of our study are mainly due to its design, that is, a retrospective study carried out in a single center. The lack of data concerning the prevalence of malaria in all sub-Saharan migrant patients attending our unit, irrespective of time after arriving in Spain and of symptomatology, is another limitation. However, in areas with large migrant populations, systematic malaria screening is expensive and is not recommended by scientific societies or international organisms. Finally, the strength of our study is that it was conducted within a specialized reference unit, allowing us to better characterize sub-Saharan immigrants diagnosed with imported malaria and to describe all associated infections.

## Conclusions

The vast majority of malaria cases in our area occur in sub-Saharan immigrants, mostly in VFR migrants who permanently reside in Spain and travel back and forth to their homeland for holidays and so on. The rise in the number of cases parallels the increase in the number of immigrants living in our area. Even though many of them have been living in Spain for a long time, < 10% report following proper malaria prophylaxis. It is a priority for health care professionals to know the peculiarities of this group of travelers, particularly the barriers to following malaria prevention measures, including malaria prevention knowledge, adherence to chemoprophylaxis, use of mosquito bite avoidance measures, and different attitudes and practices during their trips that may pose a risk to their health.

Microscopic malaria was the predominant form of malaria and fever the most frequent symptom. Nevertheless, as SMM represents almost a third of the malaria cases in our study, systematic screening might be considered, at least for more vulnerable travelers. SMM occurs in a higher proportion in non-VFR migrants, who suffer more frequently from coinfections by soil-transmitted helminths and filariae. The modulation that these pathogens exert on the immune response against malaria may play a certain protective role.

## Data Availability

The datasets analysed during the current study are available from the corresponding author on reasonable request.
